# Impact of COVID-19 Testing Strategies and Lockdowns on Disease Management Across Europe, South America, and the United States: Analysis Using Skew-Normal Distributions

**DOI:** 10.2196/21269

**Published:** 2021-04-21

**Authors:** Stefano De Leo

**Affiliations:** 1 Department of Applied Mathematics State University of Campinas Campinas Brazil

**Keywords:** COVID-19, testing strategy, skew-normal distributions, lockdown, forecast, modeling, outbreak, infectious disease, prediction

## Abstract

**Background:**

As COVID-19 infections worldwide exceed 6 million confirmed cases, the data reveal that the first wave of the outbreak is coming to an end in many European countries. There is variation in the testing strategies (eg, massive testing vs testing only those displaying symptoms) and the strictness of lockdowns imposed by countries around the world. For example, Brazil’s mitigation measures lie between the strict lockdowns imposed by many European countries and the more liberal approach taken by Sweden. This can influence COVID-19 metrics (eg, total deaths, confirmed cases) in unexpected ways.

**Objective:**

This study aimed to evaluate the effectiveness of local authorities’ strategies in managing the COVID-19 pandemic in Europe, South America, and the United States.

**Methods:**

The early stage of the COVID-19 outbreak in Brazil was compared to Europe using the weekly transmission rate. Using the European data as a basis for our analysis, we examined the spread of COVID-19 and modeled curves pertaining to daily confirmed cases and deaths per million using skew-normal probability density functions. For Sweden, the United Kingdom, and the United States, we forecasted the end of the pandemic, and for Brazil, we predicted the peak value for daily deaths per million. We also discussed additional factors that could play an important role in the fight against COVID-19, such as the fast response of local authorities, testing strategies, number of beds in the intensive care unit, and isolation strategies adopted.

**Results:**

The European data analysis demonstrated that the transmission rate of COVID-19 increased similarly for all countries in the initial stage of the pandemic but changed as the total confirmed cases per million in each country grew. This was caused by the variation in timely action by local authorities in adopting isolation measures and/or massive testing strategies. The behavior of daily confirmed cases for the United States and Brazil during the early stage of the outbreak was similar to that of Italy and Sweden, respectively. For daily deaths per million, transmission in the United States was similar to that of Switzerland, whereas for Brazil, it was greater than the counts for Portugal, Germany, and Austria (which had, in terms of total deaths per million, the best results in Europe) but lower than other European countries.

**Conclusions:**

The fitting skew parameters used to model the curves for daily confirmed cases per million and daily deaths per million allow for a more realistic prediction of the end of the pandemic and permit us to compare the mitigation measures adopted by local authorities by analyzing their respective skew-normal parameters. The massive testing strategy adopted in the early stage of the pandemic by German authorities made a positive difference compared to other countries like Italy where an effective testing strategy was adopted too late. This explains why, despite a strictly indiscriminate lockdown, Italy’s mortality rate was one of the highest in the world.

## Introduction

The study and development of models of infectious disease dynamics plays a fundamental role in the management of an unknown outbreak. Nevertheless, such models often create controversy about how, when, and whether there could be a useful tool in aiding policy decisions [1]. In the COVID-19 crisis, it appears that some articles were written to address local authorities rather than to scientifically discuss the real situation of the spread of the outbreak in each country.

It is clear that the timelier the action of local authorities, the more effective the result. The number of confirmed cases is a reliable number only if a testing strategy is adopted. Without it, we do not know in which stage of the disease the country is in at a given time. Many European countries had a similar weekly transmission rate in their *apparent* early stage of the disease, but, for example, for Italy and Spain, as well as Germany and Austria, it led to completely different outcomes. As we shall see in detail later, the massive testing strategy adopted by German and Austrian authorities created a positive difference in favor of these countries.

Often, countries are compared to each other by using their total confirmed cases. This is obviously misleading due to their varying population sizes. Nevertheless, the total confirmed cases per million (TCCpM) could also be misleading. Let us for example consider the following values taken from Worldometer [2] on May 30: Belgium, Spain, the United Kingdom, Italy, Iceland, and Singapore had a TCCpM value between 4000 and 6000. Are they in a similar situation in their management of the COVID-19 pandemic? The answer is found by examining their values for total deaths per million (TDpM), which are 815, 580, 566, 551, 29, and 4, respectively. This demonstrates clear differences in how each country was impacted by the outbreak. New Zealand, Australia, South Africa, and South Korea also have a mortality rate comparable to Singapore, but their TCCpM is approximately 300, which is well below that of Singapore. It is important to observe that, without a vaccination, *immunization* also plays a fundamental role. Hence, in the previous cases, Iceland and Singapore obtained the best results in combating the COVID-19 outbreak, whereas European countries exhibited the worst outcomes. The best way to fight the outbreak is to reach the *maximum number of immunizations together with a minimum number of deaths per million*. This point should be highlighted in scientific discussions and in the information disseminated by the media.

If a country does everything well, mortality is controlled over time. If the action of local authorities in adopting mitigation measures and testing strategies is not effective, health care systems become overwhelmed, and the mortality rate increases to critical levels. During the outbreaks in Italy and Spain, the untimely prevention and isolation measures and a weak testing strategy led to collapsed health care systems and a high mortality rate, despite lockdowns where people were only permitted to leave their homes for shopping (food and other necessities), for medical issues, and to travel to and from work only when necessary. Brazil, in time, banned international travel; canceled football matches; closed its land borders; shut down all nonessential public services (eg, all universities and primary and high schools) and private businesses, with employees working from home; and restricted commerce to supermarkets, pharmacies, restaurants (for takeaway or delivery only), gas stations, and other critical services. Despite its ineffective testing strategy when facing the outbreak, the timely action seems, at the moment, to yield good results in terms of deaths if we compare the early Brazilian stage of the disease to the European one where strict lockdowns were adopted. However, since Brazil is a big country, caution is needed when speaking of “good results.” Indeed, while some Brazilian states plan to relax the quarantine rules, others, which are facing a health system collapse, are planning, following the European example, a strict lockdown with a ban on unnecessary movement of people and vehicles.

We also find other approaches worldwide. By quickly implementing public health measures, Hong Kong demonstrated that COVID-19 transmission can be effectively contained without resorting to the strict lockdown adopted by China, the United States, and Western Europe. The Hong-Kong TCCpM is approximately 145 and the mortality rate is 0.5 (TDpM). As one of the most heavily affected epicenters during the severe acute respiratory syndrome (SARS) epidemic in 2003, Hong Kong was better equipped to face the COVID-19 outbreak compared to other countries. Improved testing, greater hospital capacity to handle novel respiratory pathogens, and a population that understood the need to improve personal hygiene and maintain physical distancing made the difference.

In Europe, one country stands out in its approach to tackle COVID-19. In Sweden, individuals took responsibility for social distancing. High schools and universities were closed, but primary schools, gyms, restaurants, and bars remained open, with social distancing rules enforced, and gatherings were restricted to 50 people. Sweden’s mortality rate per 1 million inhabitants was lower than that of Italy, Spain, and the United Kingdom but higher than its neighbors Norway, Finland, and Denmark. Nevertheless, hospitals have not, at the moment, been overwhelmed as in Italy and Spain. There is no debate over how to reopen society, and whether there will be a second wave, because society has largely remained open, and the local consequences of a lockdown have been avoided. As remarked by its local authorities, Sweden opted for a marathon-style response instead of a sprint-like one to close its first COVID-19 wave.

To understand the *mathematical* reason behind lockdowns, a brief discussion of the basic reproduction number, the so-called *R*_0_ number, is warranted [3]. It refers to the number of infected people caused by 1 infected person at the beginning of an outbreak, before widespread immunity starts to develop and/or any attempt is made to reduce transmission. The subscripted 0 refers to the lack of immunity in the population. The *R*_0_ should not be confused with *R*_t_, which is the number of persons infected, at any given time, by an infected individual. It decreases as immunized people increase, either by vaccination, natural immunity, or through death of infected persons. In the case of COVID-19, there is no vaccine as of the writing of this paper. Therefore, immunity to the infection in a large percentage of people (provided that the disease does not spread rapidly within the population), the so-called *herd immunity* [4], can only be achieved through two chains: natural immunity or death. When the number of susceptible people decreases, as people die or become immune by exposure, the *R*_t_ number decreases, and the sooner people recover or die, the smaller the *R*_t_ value becomes. The basic *R*_0_ predicts the ratio of immunization that a population requires to achieve herd immunity.

The critical immunity threshold for random vaccination (assuming 100% vaccine effectiveness) is (*R*_0_−1)*/R*_0_ [4]. For a basic *R*_0_ of 2*.*5 (the COVID-19 reproduction number estimated by Li et al [5] for Wuhan was 2.2), the critical immunity threshold is thus given by 3*/*5 (ie, 60%) of the population. For *R*_0_=5, the threshold increases to 4/5 (ie, 80%) of the population. At any time, the effective reproduction number (*R*_t_) can be expressed in terms of the *R*_0_ and the percentage of immunized people in the population at that time, *P*imm(*t*), by *R*_t_=*R*_0_[1−*P*imm(*t*)]. Mitigation and isolation strategies are often used to *artificially* reduce the reproduction number. For example, in Iran, the *R*_0_ was 4.9 in the first week [6]. After the closure of schools and universities, the *R*_t_ was 4.5, and after a reduction in work hours, this decreased to 4.3 [6].

Without a vaccine, immunization at a much-delayed speed, ensuring that health services are not overwhelmed, is the only way to manage the pandemic. Isolation (or lockdown when necessary) is the main tool to allow those experiencing the most acute symptoms to receive the medical support they need. Nevertheless, what mitigation measures should be adopted continues to be a matter of discussion; they certainly cannot be implemented without *massive testing* strategies. Indeed, testing is not only important because it shows, at any given moment, the real situation of the outbreak, it is also essential to sensitize and empower people.

A recent study from King’s College London [7], based on data from a survey of 2250 UK residents aged 18-75 years, classified the population according to their response to the COVID-19 crisis and lockdown measures. Three groups were identified: accepting (44%), suffering (47%), and resisting (9%). In the resisting cluster, with an average age of 29 years of which 64% were male, 58% thought that “too much fuss” was being made about the risk of coronavirus (around 6 times higher than in the other two groups); 76% opposed official guidelines, such as meeting friends or family outside their home (41%) or going outside when having coronavirus-like symptoms (35%). The researchers also observed that, contrary to what was observed in the resisting group, where young people dominated the sample count, people aged 55-75 years made up the largest portion of the accepting group. Women constituted nearly two-thirds of the suffering cluster, whereas men represented almost two-thirds of the resisting group. Worldwide, people spent weeks without seeing friends and/or family, without school or university, holidays, sports, or even being able to go to work. So, stress, anxiety, depression, and fear of the pandemic are common responses to lockdown measures during the COVID-19 pandemic [8,9].

## Methods

### Overview

In the early stage of the pandemic, the mitigation strategies adopted by local authorities could be monitored using countries’ weekly transmission rate. At the end of the outbreak, they can be evaluated by studying the *skew-normal* distributions that fit the daily confirmed cases and deaths curves of each country. In this paper, we analyzed in detail the testing strategies of various countries during the early stage of the COVID-19 pandemic and fitted the pandemic curves by skew-normal distributions to show how massive testing strategies are more effective than the containment measures (ie, full lockdowns) implemented in some countries.

### Data

We collated data collected by the global repositories Worldometer [2], the World Health Organization (WHO) [10], and GitHub [11].

The number of intensive care unit (ICU) beds in European countries was obtained from Rhodes et al [12]; updated counts were obtained for Germany from Brandt et al [13]. For the United States, counts were taken from Halpern and Tan [14], who reported 96,596 ICU beds (292 beds per 1 million inhabitants), with the following distribution: metropolitan, 94%; micropolitan, 5%; and rural, 1%. For Brazil, data were obtained from the Associação de Medicina Intensiva Brasileira [15]—46,000 ICU beds (216 beds per 1 million), subdivided into the five regions of Brazil: North (4%, 90 beds per 1 million), Northeast (19%, 150 beds per 1 million), Central-West (10%, 250 beds per 1 million), Southeast (52%, 270 beds per 1 million), and South (15%, 220 beds per 1 million).

### Skew-Normal Distributions

The normal distribution [16] is one of the most important probability distributions in the field of statistics because it fits many natural phenomena. It describes how the values of a variable are symmetrically distributed around its center, *μ*, and shows how the probabilities for extreme values further away from the mean go rapidly to zero in both directions. It is also known as the Gaussian distribution or the bell curve. Normal distributions are often used to fit data because, in many cases, the average point of a random variable, with a finite mean and variance, is itself a random variable whose distribution, as the number of data points increase, converges to a normal distribution. Normal distributions have also been used to fit curves pertaining to the COVID-19 pandemic. Nevertheless, their use led to misleading predictions regarding the end of the outbreak in many countries. Although we always expect uncertainties with forecasts, we must try to minimize them so that our predictions can be as close as possible to reality. It is well known that the curves of epidemiological models are *asymmetric*. So, why not use asymmetric distributions to fit the data? In particular, why do we not use skew-normal distributions in the place of normal distributions?

It is clear that before reaching the peak, normal distributions can be used to estimate the pandemic curves of daily confirmed cases per million (DCCpM) and daily deaths per million (DDpM). Indeed, eventual asymmetries can only be seen after a country has reached its peak. However, to estimate the end of the outbreak, skew-normal distributions, as we shall see later, are fundamental to obtain the correct answer. Skew-normal distributions contain an additional parameter (with respect to normal distributions) that measures the asymmetry of the curves (for a detailed review, see [17-21]). A negative value of this parameter indicates that the left tail is longer (the peak is found at the left of *μ*), and a positive one indicates that the right tail is longer (the peak moves to the right of *μ*). As seen in Multimedia Appendix 1, the blue line represents a Gaussian distribution centered at *μ*=0 (*σ*=3). The red line is a skew-normal distribution with a negative parameter (*s*=−2), and the green line represents a skew-normal distribution with a positive parameter (*s*=3).

The explicit analytical formula of the skew probabilities’ density functions, used in this paper to fit the DCCpM and DDpM curves of 12 European countries and the United States, is given by:




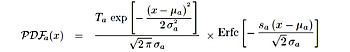




where *a=c* for the confirmed cases, *a=d* for the deaths, and *Erfc* is the complementary error function:




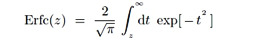




The skewness of the distribution is defined by:




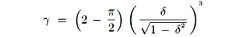




where




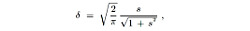




and *s* is limited to (−1*,*1). The mean value is given by *mean*=*μ+σδ,* and the mode (maximum) has not an analytic expression but, as shown by Azzalini [21], an accurate closed form, given by:




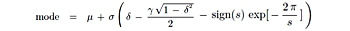




Three fitting parameters were obtained, for both the TCCpM and the TDpM data, by modeling their curves by the respective cumulative skew-normal distributions:




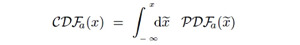




The cumulative skew-normal distribution can be expressed in terms of the complementary error function and the T-function, introduced by Owen [22] in 1956:




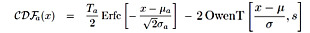




The TCCpM and TDpM curves were modeled by using the *NonlinearModelFit* calculation of the computational program Wolfram Mathematica (Wolfram Research) [23].

### The ρ Factor

Recalling that herd immunity and low mortality are *both* fundamental for tackling the outbreak, we introduce the *ρ* factor, which can be used to easily compare countries. If two countries have the same TCCpM value, the one with the greater tests per confirmed case (TpC) value should have a lower number of infected people in its population with respect to the other. A lower *ρ* value implies a better rating:




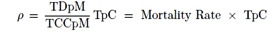




## Results

### Mortality Rate

Based on data collected from global repositories, Table 1 displays statistics for 12 European countries, 10 South American countries, and the United States, as of May 30, 2020.

On May 30, 2020, the total death count was greatest for Italy (TDpM=551.1), the United Kingdom (TDpM=566.0), Spain (TDpM=579.6), and Belgium (TDpM=814.9). In these countries, the TpC number was similar (14.9 for Belgium and Spain, 15.3 for the United Kingdom, and 16.4 for Italy), and their TCCpM ranged from 3845.7 (Italy) to 5111.7 (Spain). The mortality rate was 16.2% for Belgium, 11.3% for Spain, 14.3% for Italy, and 14.1% for the United Kingdom. Ireland and Switzerland, which had a TpC ratio of 13.0 and 12.8, had a lower mortality rate (6.6% and 6.2%, respectively). The United Kingdom and Ireland had a similarly low number of ICU beds (the WHO suggests a number between 100 and 300 beds per 1 million population as adequate) but a differing mortality rate. The same occurred for Italy (125 beds per million) and Spain (97 beds per million), and Switzerland (110 beds per million). Belgium, despite an adequate number of beds per million (159), had the worst mortality rate.

Sweden and the Netherlands had a TpC ratio of 6.2 and 7.1, respectively; that of the United States was 9.1. For these countries, the mortality rate was 11.8% (Sweden), 12.8% (Netherlands), and 5.9% (United States). Here, the great difference in the number of ICU beds and the temporal shift at the beginning of the outbreak (allowing for better preparation of the health care system) clearly played a fundamental role.

**Table 1:** The total deaths per 1 million inhabitants, the total confirmed cases per million, tests per confirmed case, population size in millions, population density per km2, and the number of intensive care unit (ICU) beds per million for 12 European countries, 10 South American countries, and the United States, as of May 30, 2020.

Country	Total deaths per million	Total confirmed cases per million	Tests per confirmed case	Population size in millions	Population density	ICU beds per million
Belgium	814.9	5016.0	14.9	11.6	376	159
Spain	579.6	5111.7	14.9	46.8	96	97
United Kingdom	566.0	4024.0	15.3	67.8	274	66
Italy	551.1	3845.7	16.4	60.5	200	125
France	439.8	2842.5	7.5	65.3	119	116
Sweden	435.1	3674.6	6.4	10.1	23	58
Netherlands	348.0	2705.1	7.5	17.1	421	64
Ireland	336.9	5087.6	13.0	4.9	70	65
United States	313.7	5351.2	9.8	330.8	36	292
Switzerland	223.1	3586.6	12.8	8.6	208	110
Ecuador	189.4	2191.5	2.9	17.6	63	N/A^a^
Portugal	136.9	3157.2	24.7	10.2	112	42
Brazil	135.8	2346.7	1.9	212.4	25	216
Peru	132.9	4731.6	6.5	32.9	25	N/A
Germany	101.8	2186.0	21.6	83.8	233	339
Austria	74.2	1853.9	26.9	9.0	76	218
Chile	52.2	4966.4	5.9	19.1	23	N/A
Bolivia	26.5	819.8	3.0	11.7	10	N/A
Columbia	17.5	526.3	12.0	50.8	41	N/A
Argentina	11.7	359.5	9.6	45.1	16	N/A
Uruguay	6.3	234.6	53.3	3.5	20	N/A
Paraguay	1.5	135.8	30.1	7.1	17	N/A
Venezuela	0.5	51.4	669.0	28.4	35	N/A

^a^N/A: not applicable.

For Brazil, which had the lowest TpC value (1.9), the mortality rate was 5.8%, similar to the United States. It is clear that for all the countries, due to the fact that there was a good number of asymptomatic people, an increasing number of tests should decrease the mortality rate—that is, when the TpC number resembles Spain’s value, the mortality rates of Sweden, the Netherlands, the United States, and Brazil should further decrease. Portugal (TpC=24.7), Germany (TpC=21.6), and Austria (TpC=26.9) had the largest TpC numbers and exhibited a very low mortality rate of 4.3%, 4.7%, and 4.0%, respectively.

It should be noted that when comparing the mortality rate percentage, we must consider the number of tests done per confirmed case. To correctly interpret any data, we need to know how much testing for COVID-19 has been done by the country. Without complete data, it becomes difficult to assess which countries are doing well and understand how the pandemic is spreading. When discussing the total deaths per 1 million population, the number of tests is not important. In this case, we have to consider the stage of the outbreak. For example, the South American countries are in a stage of infection different to that of the European countries, which are closing their first COVID-19 wave. Looking at the total deaths per 1 million population, a particular case is called to our attention. In Table 1, of the first 4 countries listed, Italy had the highest TpC number (16.4), and the value for Germany was 21.6. Considering that both countries are closing their first wave of the pandemic, how can their large difference in TDpM (Italy: 551.1 vs Germany: 101.8) be justified?

To answer to this question, we looked at the data reported in Table 2 and collected for Austria, Germany, and the 4 countries with the greatest TDpM numbers in Table 1 (Belgium Spain, Italy, and the United Kingdom) according to the Our World in Data repository [24].

**Table 2:** Tests per million, total confirmed cases per million, and tests per confirmed case for Austria, Germany, Italy, the United Kingdom, Spain, and Belgium on different dates.

Country and date			Tests per million		Total confirmed cases per million		Tests per confirmed case
**February 27**							
	Austria	50		0.33		151.5	
	Italy	200		10.83		18.5	
**March 8**							
	Austria	500		11.56		43.3	
	Germany	1490		12.41		120.1	
	Italy	830		121.9		6.8	
	Belgium	350		17.24		20.3	
**March 15**							
	Austria	910		95.56		9.5	
	Germany	3010		69.15		43.5	
	Italy	2070		409.04		5.1	
	Belgium	1070		76.38		14.0	
**March 22**							
	Austria	2370		398.00		6.0	
	Germany	7170		296.81		24.2	
	Italy	4270		977.49		4.4	
	Belgium	2220		293.19		7.6	
**March 29**							
	Austria	5160		976.44		5.3	
	Germany	11,490		740.99		15.5	
	Italy	7510		1614.69		4.7	
	Belgium	4000		934.14		4.3	
**April 5**							
	Austria	12,040		1339.00		9.0	
	Germany	16,360		1194.79		13.7	
	Italy	11,440		2131.37		5.4	
	Belgium	6320		1697.5		3.7	
**April 13**							
	Austria	16,480		1560.11		10.6	
	Germany	20,890		1552.17		13.5	
	Italy	17,320		2636.63		6.6	
	United Kingdom	5420		1307.09		4.1	
	Spain	19,900		3634.59		5.5	
	Belgium	9900		2636.98		3.8	

### Massive Testing Strategy

On March 8, Belgium, Austria, and Germany had a similar TCCpM value (between 10 and 20) but a different TpC number: 20.3, 43.3, and 120.1, respectively. This indicates that when the pandemic was in its initial stage reaching the TCCpM value of 10-20, the testing strategy in Austria was twice as effective as that of Belgium, and Germany showed a massive testing strategy 6 times more effective than Belgium and twice as effective compared to Austria. On March 8, the pandemic in Italy was at an advanced stage, with a TCCpM value of 121.9.

To compare the testing strategy of Italy with that of Germany, we have to go back to February 27 when Italy’s TCCpM was 10.83. The TpC number of Italy, when the disease reached 10-20 TCCpM, was similar to Belgium. An easy way to compare testing strategies is by normalizing the TpC to one of the compared countries. This allows us to yield an effectiveness factor (EF) with respect to that country. For example, by choosing Italy as the normalizing country, the EF for Belgium, Austria, and Germany is 1.1, 2.3, and 6.5, respectively. Table 3 reports the EFs generated when repeating this for other intervals of TCCpM.

**Table 3:** The effectiveness factor of the testing strategy of Italy, Belgium, Austria, and Germany.

Intervals of total confirmed cases per million	Effectiveness factor			
	Italy	Belgium	Austria	Germany
10-20	1.00 (18.5)	1.10 (20.3/18.5)	2.34 (43.3/18.5)	6.49 (120.1/18.5)
250-450	1.00 (5.1)	1.49 (7.6/5.1)	1.18 (6.0/5.1)	4.75 (24.2/5.1)
900-1200	1.00 (4.4)	0.98 (4.3/4.4)	1.20 (5.3/4.4)	3.11 (13.7/4.4)
1300-1700	1.00 (4.7)	0.78 (3.7/4.7)	1.91 (9.0/4.7)	2.87 (13.5/4.7)

**Figure 1:**
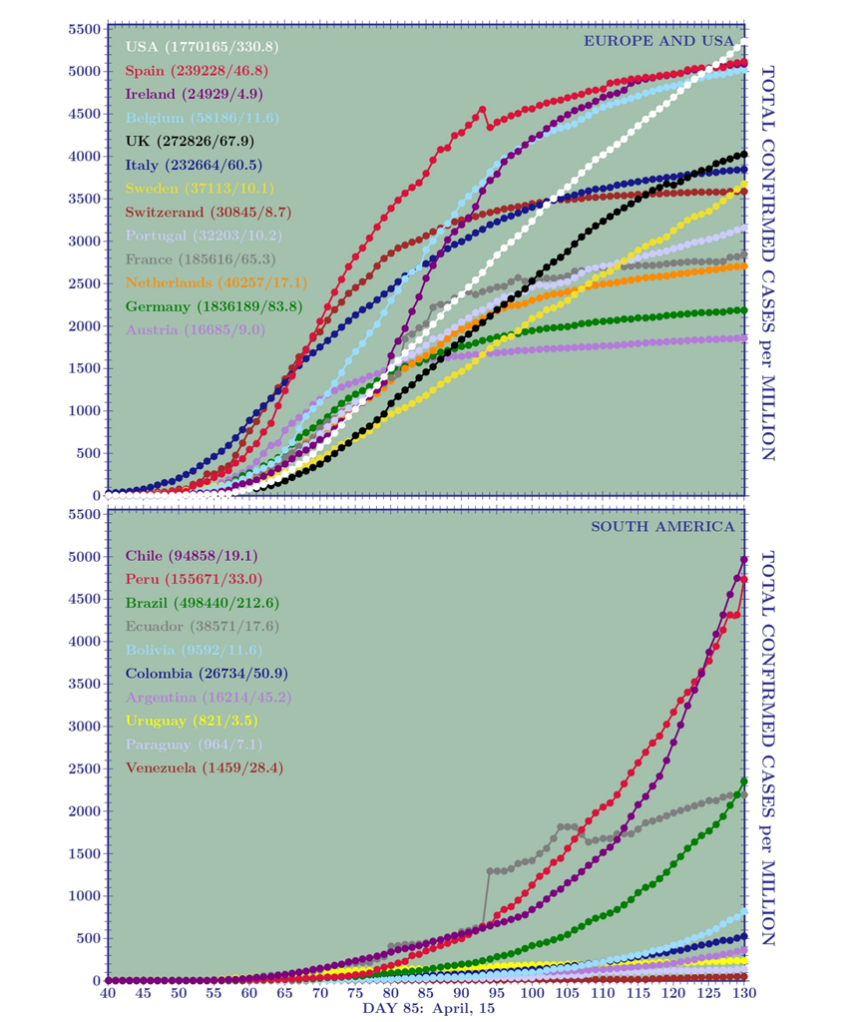
Curves of the total confirmed cases per 1 million inhabitants (TCCpM) for (A) 12 European countries and the United States and (B) all South American countries, on day 130 (May 30, 2020). A stabilization point is seen in almost all European countries. This has not yet occurred in South America where the outbreak is delayed with respect to Europe. Among the 12 European countries analyzed, the higher TCCpM numbers belong to Spain, Ireland, and Belgium, followed by Italy and Switzerland. The United States overtook the European countries with the highest TCCpM numbers, the United Kingdom overtook Italy, and Sweden sits between Switzerland and Italy.

**Figure 2:**
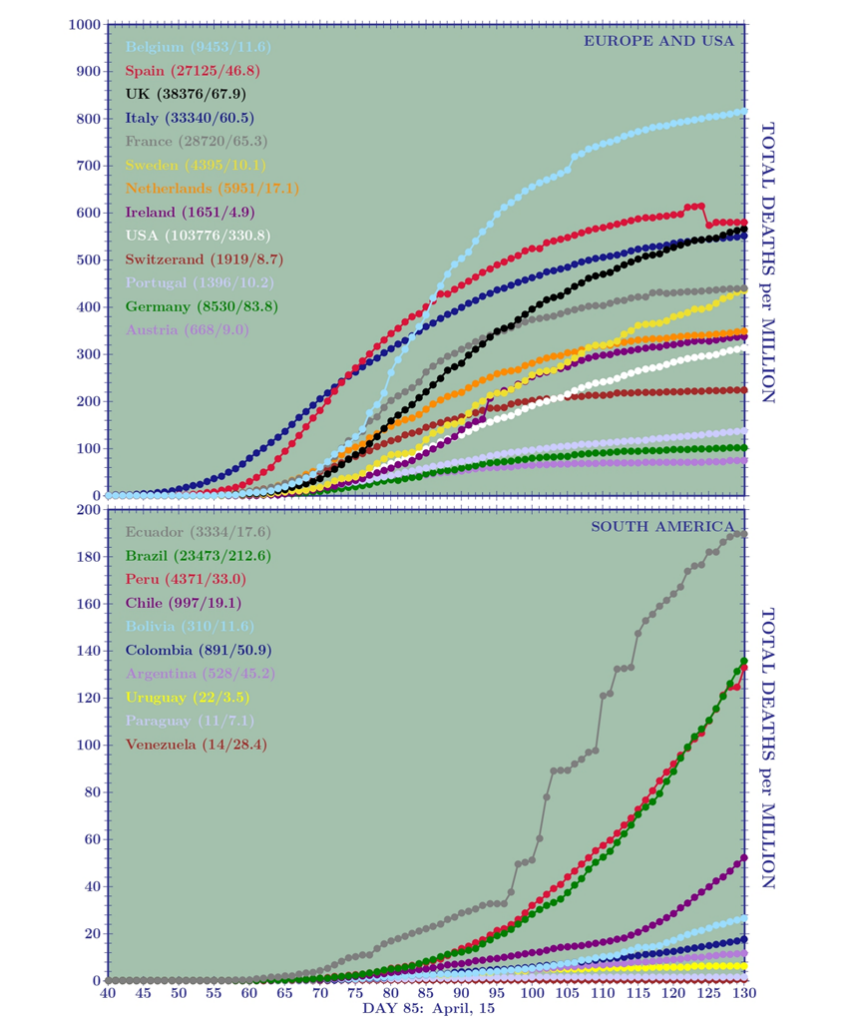
Curves of the total deaths per 1 million inhabitants (TDpM) for (A)12 European countries and the United States and (B) all South American countries, on day 130 (May 30, 2020). Among the 12 European countries analyzed, the higher TDpM numbers belong to Belgium, Spain, the United Kingdom, and Italy. The Spain anomaly is due to the lower number of deaths on day 125 (26,837) with respect to deaths on day 124 (28,752). Among the South American countries, Ecuador shows the more critical situation, followed by Peru and Brazil with nearly the same number of deaths per million and very similar curves.

In Figures 1 and 2, we show the temporal behavior of the TCCpM and TDpM curves for the United States and for countries in Europe and South America. The data of Table 1 and the plots of Figures 1 and 2 are periodically updated online [25].

Lastly, it is worthwhile to discuss the situation in Venezuela (Table 1, last row), whose TDpM was 0.5, TCCpM was 51.4, and TpC was surprisingly 669. Due to its socioeconomic and political crisis, Venezuela was isolated from the world even before the COVID-19 outbreak and was the first nation in South America to impose a strict lockdown. This may explain the lack of widespread transmission in Venezuela. With respect to the high number of tests, it is important to observe that Venezuela performed a substantial number of rapid blood antibody tests (manufactured in China) checking for proteins developing after someone is infected [26]. Few nasal swab exams were used by local authorities. It is important to recall that only swab-test positives are added to the official statistics of confirmed cases. Inclusion or exclusion of antibody tests explains why, for example, the total number of confirmed cases reported for Spain by Worldometer [2], where antibody tests are considered, and in GitHub [11], where they are not, differ.

### Weekly Transmission Rates

We now discuss the weekly rate of DCCpM and DDpM. Before introducing what, for simplicity, we refer to as alpha (*α)* [27] and beta *(β)* factors, we first compare the outbreak in different countries. We shall analyze, as an illustrative example, Germany and Italy, the United States, and Brazil. In these countries, the outbreak did not start at the same time. Hence, we compared them with each other to see when they reached the same number of TCCpM. Let us consider the moment at which they reached 10 TCCpM. This happened for Italy on February 27 (TCCpM=10.83), for Germany on March 7 (TCCpM=9.53), for the United States on March 15 (TCCpM=10.69), and, finally, for Brazil on March 24 (TCCpM=10.58). To see how the outbreak was spreading in these countries, we can compare their DCCpM numbers. This can be done by averaging the weekly data from February 27 for Italy, March 7 for Germany, March 15 for the United States, and March 24 for Brazil. This comparison can be also done for a TCCpM value of 100 (Table 4).

**Table 4:** The weekly transmission rate of daily confirmed cases per million and tests per million for Italy, Germany, the United States, and Brazil upon reaching 10 and 100 total confirmed cases per million.

Country			Total confirmed cases per million	Date		7-day moving average^a^		Tests per million
***α* factor**								
	Italy	10.83		Feb 27	3.63		200	
	Germany	9.53		Mar 7	2.15		1490	
	United States	10.69		Mar 15	2.81		120	
	Brazil	10.58		Mar 24	1.76		N/A^b^	
***β* factor**								
	Italy	97.24		Mar 7	18.06		700	
	Germany	110.47		Mar 17	27.57		3010	
	United States	100.61		Mar 22	25.07		760	
	Brazil	97.58		Apr 11	7.55		300	

^a^Daily confirmed cases per million/7.

^b^N/A: not applicable.

**Figure 3:**
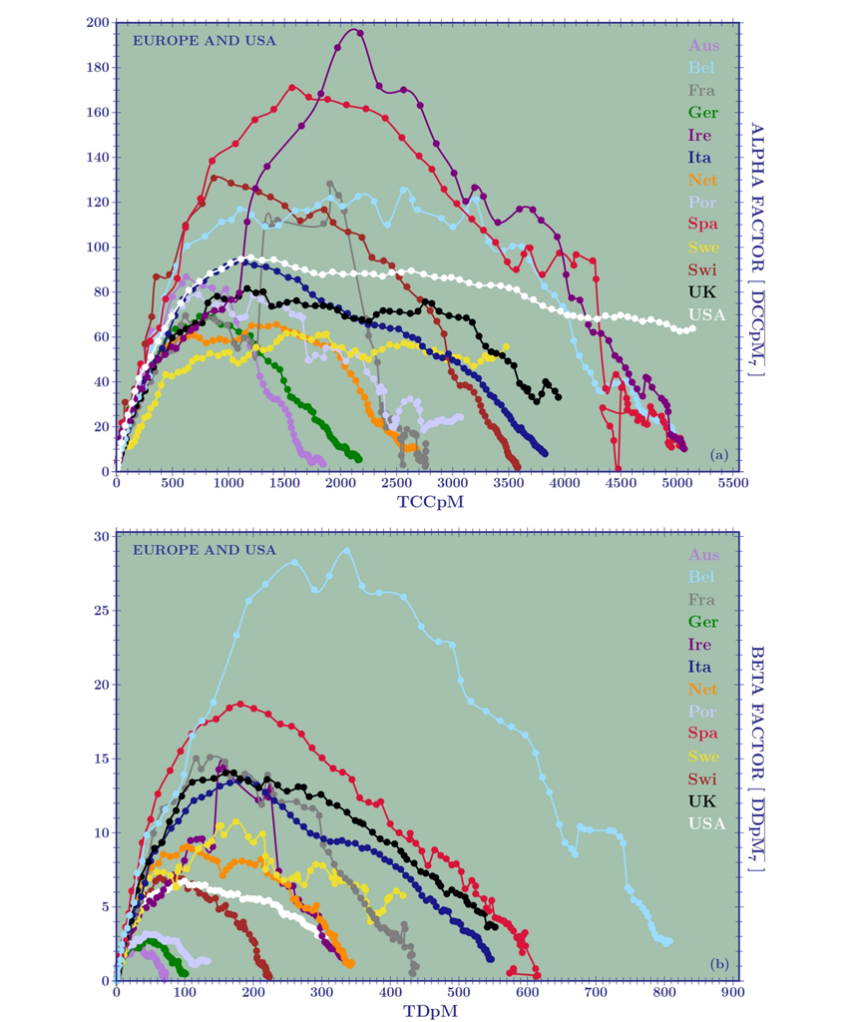
The weekly spreading rate for (A) daily confirmed cases per million (DCCpM; α factor) and (B) daily deaths per million (DDpM; β factor), calculated for 12 European countries and the United States when these countries reach the same value for total confirmed cases (TCCpM) and total deaths per million (TDpM). For the factor, the number of tests per million should be considered as normalization, but this number is not always available. The curves show a clear asymmetry. They allow for the prediction of a final TCCpM greater than 5000 for Ireland, Spain, and Belgium; around 4000 for Italy and the United Kingdom; and around 2000 for Austria and Germany. For total deaths, Belgium exhibited the worst result (around 800), followed by Spain, the United Kingdom, and Italy (around 600). Austria and Germany had lower mortality rates.

**Figure 4:**
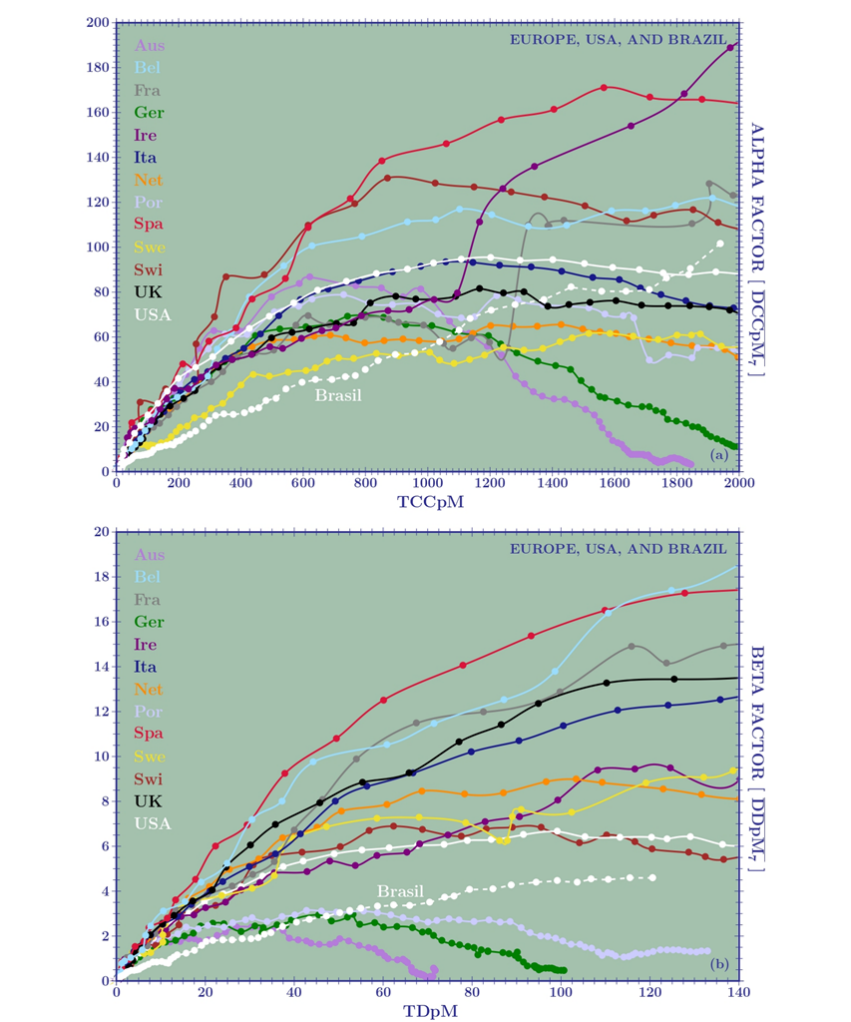
The weekly spreading rate at the beginning of the outbreak for 12 European countries, the United States, and Brazil. (A) Confirmed cases: Brazil, with an initial behavior similar to Sweden, shows a steep increase in its curve, overtaking most European countries and the United States. (B) Deaths: the Brazilian curve overtakes those of Austria, Germany, and Portugal (which have the lowest mortalities) but remains below all other European countries and the United States. DCCpM: daily confirmed cases per million, DDpM: daily deaths per million, TCCpM: total confirmed cases per million, TDpM: total deaths per million.

Figure 3A is a plot of the *α* factor for 12 European countries and the United States. The weekly transmission rate of DCCpM were greatest for Ireland and Spain (~200 and 180, respectively), followed by Belgium and Switzerland (both ~130), with the first three countries closing their first wave of the pandemic with a TCCpM around 5000. Italy and Germany showed a maximum rate of approximately 100 and 70, respectively, and a final TCCpM of around 4000 and 2000, respectively. Figure 4A demonstrates that all the European countries, with the exception of Sweden, present the same curves for their initial weekly transmission rate. In particular, the *α* factor of the United States followed, up to 1000 TCCpM, the same curve as Italy. So, why do the European countries exhibit a different behavior in the successive stages of the outbreak?

The answer once again comes from the testing strategy adopted by local authorities and can be seen by observing Table 4. Italy (on February 27) and the United States (on March 15) reached TCCpM values of 10.83 and 10.69, respectively, with an *α* factor of 3.63 for Italy and 2.81 for the United States. Due to the fact that, at that time, Italy and the United States tested 200 and 120 inhabitants per million, respectively, their initial-stage behavior was comparable. The plots in Figure 3A, as well the amplification done in Figure 4A, are not normalized. Hence, Germany’s curve is similar to those of Italy and the United States. Nevertheless, looking at the last column of Table 4, we immediately see a great difference in the testing strategy of Germany (1490 tests per million) compared to Italy (200 tests per million) and the United States (120 tests per million), leading to a German relative factor with respect to Italy of (2*.*15/3.63) × (200*/*1490) ≈ 0*.*29/3*.*63, and to the United States of 0*.*17/2*.*81*.* Reaching 100 TCCpM, the German effective factors become 6*.*41/18*.*06 and 6*.*96/25*.*07.

Data on the testing strategy adopted by the different countries are often available. Hence, when the plots given in Figures 3A and 4B are used to compare countries to each other, they have to be appropriately normalized by the tests per million relative ratio.

We recall one more time that the success of a country in combating the pandemic is not to reduce the TCCpM but to reduce its TDpM. Immunization also plays a fundamental role in disease management. Obviously, reducing infections also has an effect on decreasing the rate of mortality. However, it is possible to find many examples in which a large TCCpM value does not necessarily imply a large TDpM value (see, for example, Ireland’s curves in Figure 3).

Next, we analyzed the weekly transmission rate for DDpM, the so-called *β* factor, which was done analogously to what has been done for the DCCpM. Table 5 takes Italy, Germany, the United States, and Brazil as illustrative examples.

In this case, the comparison can be done directly without any testing normalization. Obviously, subnotification of deaths has to be considered as well, but, at the moment, we have no reliable information on this. Between 10 and 20 TDpM, Table 5 shows the worst *β* factors for Italy and the best ones for Brazil. Nevertheless, the increasing rate for Italy, Germany, the United States, and Brazil show a factor of 1.6, 1.4, 1.6, and 2.1, respectively. In Figure 3B, we see that Ireland, despite its high values for TCCpM and peak in DCCpM, will close its first wave of the pandemic with a TDpM value between 300 and 400, well below Belgium (TDpM=800) and Italy, Switzerland, and Spain (TDpM range 550-650). The plots also show good results for Austria (TDpM*<*100), Germany (TDpM~100), and Portugal (TDpM=150).

In Figure 4B, which is an amplification of Figure 3B, Brazil overtakes the curves of Austria, Germany, and Portugal (meaning that its final TDpM will be greater than 200) but is still under that of other European countries and the United States.

**Table 5:** The β factor for Italy, Germany, the United States, and Brazil upon reaching 10 and 20 total deaths per 1 million population.

Country		Total deaths per million	Date	7-day moving average^a^
**10 total deaths per million**				
	Italy	10.43	Mar 10	2.52
	Germany	10.98	Apr 1	1.72
	United States	10.34	Mar 29	2.24
	Brazil	10.08	Apr 17	0.85
**20 total deaths per million**				
	Italy	20.93	Mar 13	4.00
	Germany	18.90	Apr 5	2.44
	United States	19.68	Mar 1	3.51
	Brazil	20.18	Apr 17	1.75

^a^Daily total deaths/7.

### Analysis of Skew-Normal Distributions

**Figure 5:**
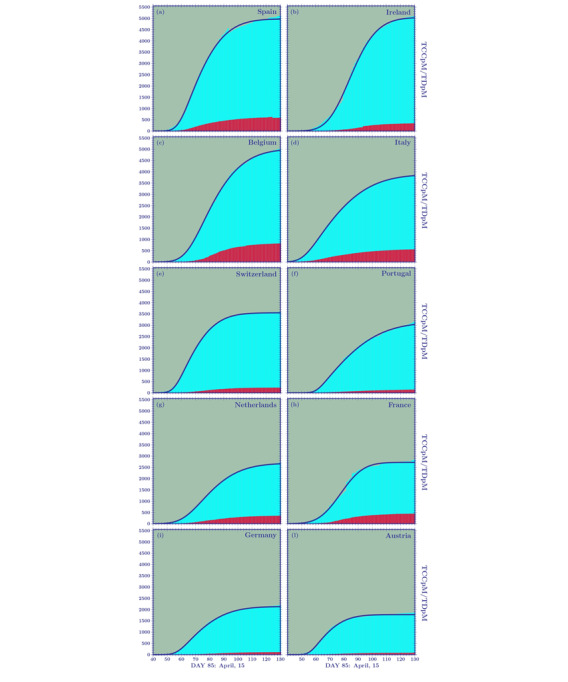
Skew-normal cumulative distribution functions for 10 European countries that have closed their first pandemic wave. TCCpM: total confirmed cases per million, TDpM: total deaths per million.

**Figure 6:**
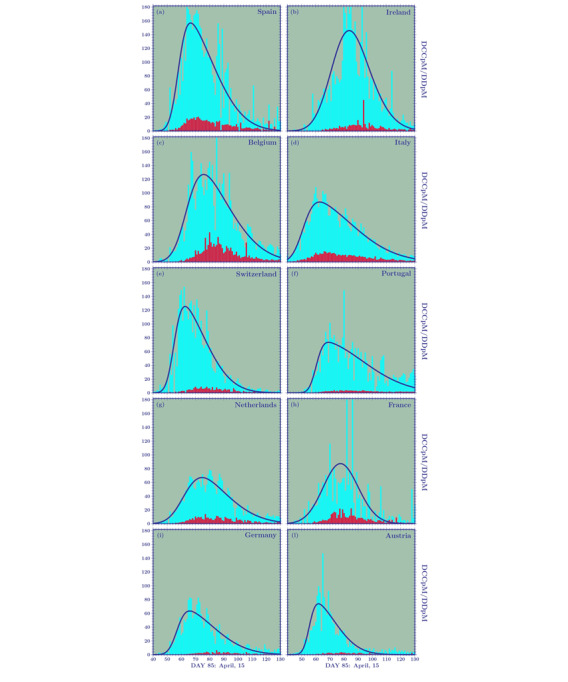
Skew-normal probability distribution functions corresponding to the cumulative distribution functions plotted in Figure 5. DCCpM: daily confirmed cases per million, DDpM: daily deaths per million.

The three fitting parameters, with their respective 95% CIs, are shown in Tables 6 and 7 for 10 European countries. The cumulative density function and probability density function for these countries, which are closing their first pandemic wave, are displayed in Figures 5 and 6. The DCCpM plots in Figure 6 clearly show their asymmetric nature. This explains why forecasts based on normal distributions, due to the lack of profile asymmetry, leads to misleading results.

**Table 6:** The fitting parameters (center, standard deviation, and skewness) of the skew-normal distributions for the countries in Figures 5 and 6 for total confirmed cases per million.

Country	Parameter				Total confirmed cases per million (95% CI)
	*μ*_c_(95% CI)	*sig*_c_ (95% CI)	*s*_c_ (95% CI)		
Ireland	73.4 (1.4)	19.2 (0.1)	1.6 (0.3)	5040 (39)	
Belgium	63.7 (0.4)	26.5 (0.1)	3.3 (0.3)	5014 (25)	
Spain	57.8 (0.5)	22.6 (0.1)	4.6 (0.8)	4977 (26)	
Italy	50.9 (0.2)	32.3 (0.1)	5.1 (0.3)	3889 (10)	
Switzerland	54.8 (0.2)	20.1 (0.1)	4.5 (0.5)	3551 (10)	
Portugal	60.2 (0.4)	32.4 (0.1)	8.2 (2.2)	3133 (32)	
France	70.8 (6.2)	14.4 (0.3)	0.8 (0.9)	2723 (24)	
The Netherlands	62.1 (0.4)	25.9 (0.1)	2.8 (0.2)	2684 (12)	
Germany	56.9 (0.4)	24.3 (0.1)	5.0 (0.9)	2136 (11)	
Austria	55.4 (0.6)	17.3 (0.1)	4.9 (1.7)	1777 (11)	

**Table 7:** The fitting parameters (center, standard deviation, and skewness) of the skew-normal distributions for the countries in Figures 5 and 6 for total deaths per million.

Country	Parameter				Total deaths per million (95% CI)
	*μ*_d_ (95% CI)	*sig*_d_ (95% CI)	*s*_d_ (95% CI)		
Belgium	71.2 (0.4)	21.8 (0.5)	3.7 (0.4)	810 (4)	
Spain	59.8 (0.4)	26.1 (0.6)	7.0 (1.6)	600 (4)	
Italy	54.3 (0.2)	33.7 (0.3)	5.8 (0.4)	562 (2)	
France	66.6 (0.4)	22.6 (0.6)	4.4 (0.6)	436 (2)	
The Netherlands	64.8 (0.2)	28.0 (0.4)	4.3 (0.3)	354 (1)	
Ireland	82.3 (2.6)	16.1 (2.0)	1.2 (0.5)	330 (4)	
Switzerland	64.7 (0.2)	21.8 (0.3)	3.2 (0.2)	223 (1)	
Portugal	66.2 (0.4)	34.8 (1.1)	5.9 (0.9)	143 (2)	
Germany	70.4 (0.4)	24.9 (0.6)	3.1 (0.3)	102 (1)	
Austria	66.9 (0.4)	20.2 (0.5)	2.8 (0.3)	71 (1)	

The greatest asymmetries are found in the skew-normal distributions of Portugal for confirmed cases (γ*_c_*=0*.*94) and of Spain for deaths (γ*_d_=*0*.*92). The most symmetric distributions belong to Ireland (γ*_c_=*0*.*33 and γ*_d_=*0*.*20) and France (γ*_c_=*0*.*08), each with a profile very similar to Gaussian distributions.

By using the fitting parameters of the skew-normal distributions, we can also obtain information about the mean values of the DCCpM and DDpM curves. For example, for Germany, Spain, Italy, and Belgium, we find *μ_c_=*75*.*9*,* 75*.*4*,* 76*.*1*,* and 83*.*9, respectively, showing that the epidemic began in the same period in the first three countries and a week later in Belgium. It is also interesting to calculate the shift between the mean values of deaths and confirmed cases (Δmean=*μ_d_–μ_c_+σ_d_δ_d_–σ_c_δ_c_).* For Germany, this value was 13.4. For Spain, Italy, and Belgium, it is lower: 5.0, 4.7, and 2.5, respectively. This indicates that in Spain, Italy, and Belgium, only people with moderate or severe symptoms were being tested; this serves as additional evidence of the different testing strategies adopted in the early stage of the outbreak by Spain, Italy, and Belgium vs Austria and Germany.

We observed that, among the distributions plotted in Figure 6, that of the Netherlands shows a smooth growth and a peak (comparable to that of Germany) and is lower than all the other distributions. The Netherlands attempted to adopt a different form of lockdown. In contrast to most other European countries, where people were virtually housebound, the Dutch authorities opted for what they called an “intelligent” lockdown. The Dutch position, in many aspects similar to the Swedish one, reflects the idea that immunization also plays a fundamental role in managing the pandemic. Despite its differing approach with respect to the strict lockdowns of Belgium (TDpM=814.9), Spain (TDpM=579.6), the United Kingdom (TDpM=566.0), Italy (TDpM=551.1), and France (TDpM=439.8), the Netherlands seems to have made the right choice, closing their first wave of the outbreak with a smaller number of deaths per million (TDpM=348.0).

#### The United Kingdom, Sweden, the United States, and Brazil

**Figure 7:**
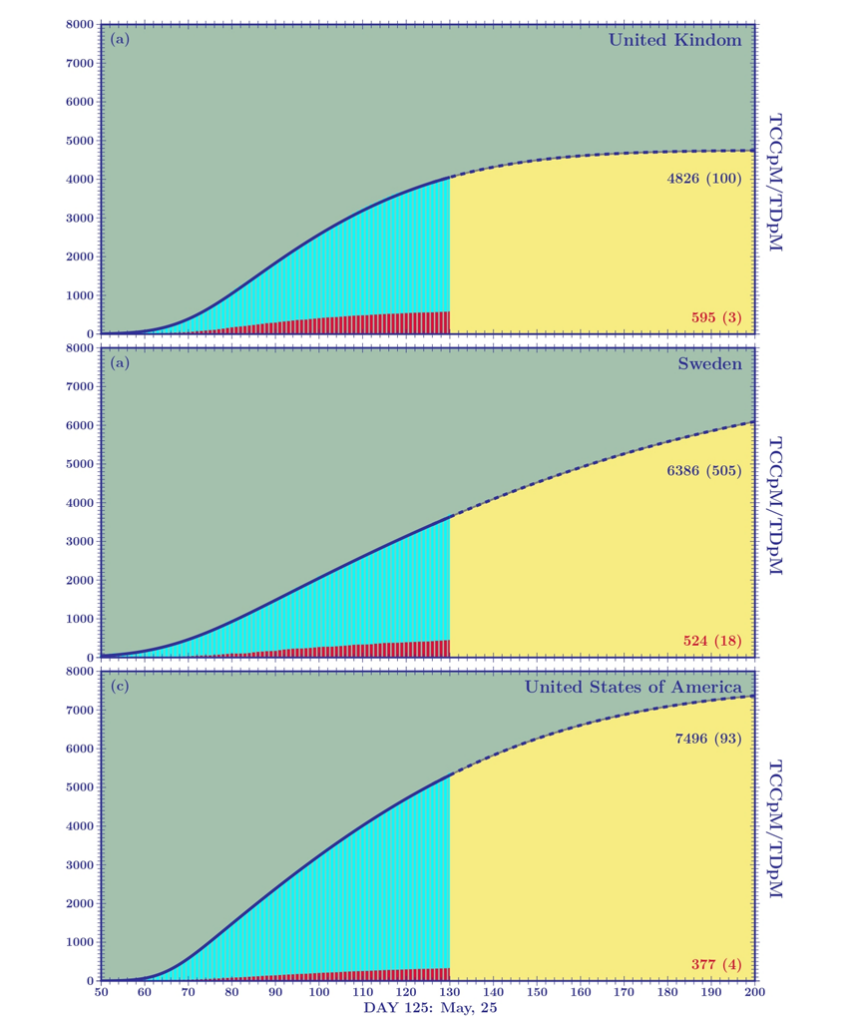
Skew-normal cumulative distribution functions for the United Kingdom, Sweden, and the United States.

**Figure 8:**
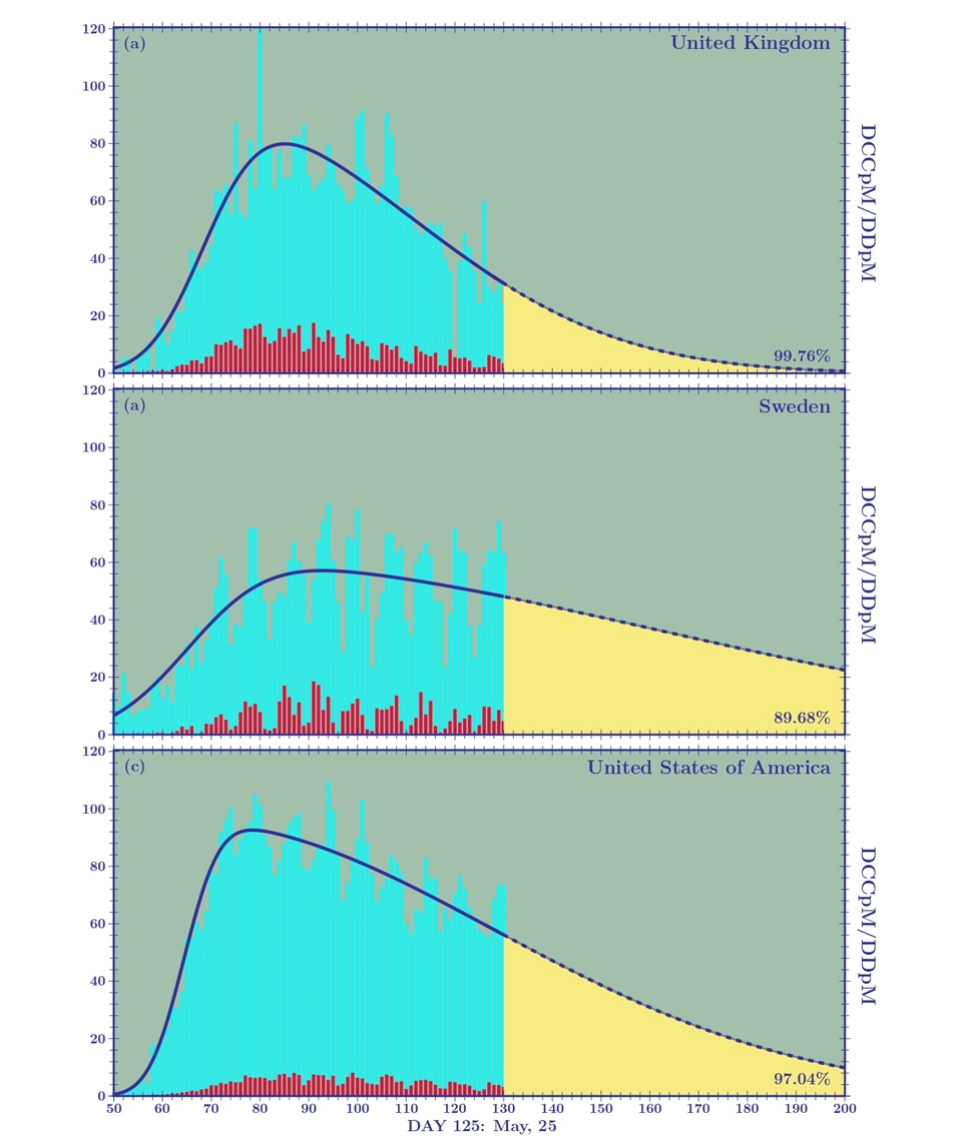
Skew-normal probability distribution functions corresponding to the cumulative distribution functions plotted in Figure 7. DCCpM: daily confirmed cases per million, DDpM: daily deaths per million.

In Figures 7 and 8, we plot the cumulative density function and probability density function skew-normal distributions for the United Kingdom, Sweden, and the United States. The fitting parameters modeling the TCCpM and TDpM curves are given in Tables 8 and 9.

**Table 8:** The fitting parameters (center, standard deviation, and skewness) of the skew-normal distributions of the United Kingdom, Sweden, and the United States for total confirmed cases per million.

Country	Parameter				Total confirmed cases per million (95% CI)
	*μ*_c_(95% CI)	*sig*_c_ (95% CI)	*s*_c_ (95% CI)		
United States	64.4 (0.1)	63.6 (0.1)	11.1 (0.6)	7618 (90)	
Sweden	65.3 (0.6)	95.8 (1.1)	7.6 (1.2)	7253 (675)	
United Kingdom	68.6 (0.4)	42.4 (0.1)	4.6 (0.4)	4753 (78)	

**Table 9:** The fitting parameters (center, standard deviation, and skewness) of the skew-normal distributions of the United Kingdom, Sweden, and the United States for total deaths per million.

Country	Parameter				Total deaths per million (95% CI)
	*μ*_d_ (95% CI)	*sig*_d_ (95% CI)	*s*_d_ (95% CI)		
United States	69.5 (0.2)	31.9 (0.5)	5.4 (0.4)	595 (4)	
Sweden	72.3 (0.6)	43.5 (2.8)	5.7 (1.0)	524 (18)	
United Kingdom	70.6 (0.2)	43.2 (0.8)	6.4 (0.4)	377 (4)	

The curves of the United Kingdom and Sweden, in terms of the DCCpM and DDpM skewness and DDpM standard deviation, are similar to that of Italy and Portugal, respectively. The difference is found in the standard deviation of DCCpM. The *c value* for the United Kingdom (*c*=42.4) is greater than that of Italy (*c*=32.3), and the *c* of Sweden (*c*=95.8) is the highest among all the countries studied in this paper. Sweden’s high standard deviation is a clear consequence of the milder mitigation measures adopted by local authorities. Contrary to what will happen in other European countries, where once the first phase of the pandemic is closed and a new wave is expected to come, Sweden will probably face a single long period of the pandemic.

The greater standard deviations of the DCCpM curves of the United Kingdom and Sweden, with respect to those pertaining to their DDpM, leads to mean values of DDpM lower than those of DCCpM (United Kingdom: *μ_d_=*94*.*5, *μ_c_=*101*.*7; Sweden: *μ_d_=*106*.*5, *μ_c_=*141*.*1), which is contrary to what has been seen for other European countries. This result confirms what we discussed in the *Introduction*, that is, when speaking of COVID-19 numbers, it is fundamental to look at the deaths per million. Predictions of the critical peak region for the DDpM curves are clearly more important than the ones for the DCCpM curves. When the DDpM curves cannot be modeled, because one of the three parameters oscillates, we can resort to what we call dynamical prediction. This happens, for example, for Brazil, where the peak still shows an oscillating behavior. This point will be revisited later.

The skew-normal predictions can be complemented by the graphical analysis of the *α* and *β* factors given in Figure 3 . For example, Figure 3A shows a closing curve for the United Kingdom (black line) between 4000 and 5000 TCCpM, and this is in agreement with the skew-normal prediction (4753, SD 78). For the United Kingdom, with a population of 68 million people, a TCCpM of 5000 means 340,000 confirmed cases at the end of the first pandemic wave. For Sweden (yellow line), the factor does not yet show a decreasing trend. This means that the skew-normal forecast yields a TCCpM value greater that 7000 (7253, SD 675) corresponding to 70,000 confirmed cases (considering that the Swedish population is 10 million inhabitants), which could represent a lower limit. As observed before, the number of total infected people is only one of the analyses that needs to be done to assess how a country has tackled the epidemic. When looking at the skew-normal predictions for the total deaths in the United Kingdom and Sweden, we find values around 600 (595, SD 4) and 500 (524, SD 18), respectively. This predicts approximatively 40,000 and 5000 deaths for the United Kingdom and Sweden, respectively.

For the United States, the skew-normal prediction for the TCCpM results in a value of approximately 7500 (7618, SD 90); this means that for a population of 330 million people, there will be 2.5 million confirmed cases at the end of the first pandemic wave. Interestingly, the TCCpM of the United States and Sweden is similar despite differing mitigation measures. However, as observed earlier, when we compare the total confirmed cases between two countries, we must normalize using their TpC ratio, which in this case is 2/3 (Table 1).

The United States and the United Kingdom similarly adopted strict lockdowns. The factor of the United States (Figure 3B, white line) predicts, at the end of the first wave, a TDpM of 400 (130,000 deaths) compatible with the skew-normal prediction (377, SD 4). The United Kingdom should close its first wave with a TDpM of 600. This difference could be explained by the difference in the number of ICU beds per 1 million for the two countries (66 for the United Kingdom and 292 for the United States). Sweden, if the prediction is confirmed, should close with a TDpM of 500 without resorting to a strict lockdown and despite its very low number of beds per 1 million (58), which is certainly a win for the Swedish authorities. It should be noted that most European countries are now entering the second phase of COVID-19, and as mitigation measures are relaxed, their response will resemble the Swedish approach.

For Brazil, it is not yet possible to model the DCCpM and DDpM curves because the skew-normal parameters are still in their oscillating phase. However, the α and *β* factors can be used to compare the epidemic curves of Brazil with those of the European countries when they were in the same stage of the outbreak. In particular, the Brazilian DDpM weekly spreading curve (Figure 4B) overtakes those of Austria, Germany, and Portugal, but it is lower than those of other European countries like Spain, Italy, and the United Kingdom.

### Dynamical Predictions

**Figure 9:**
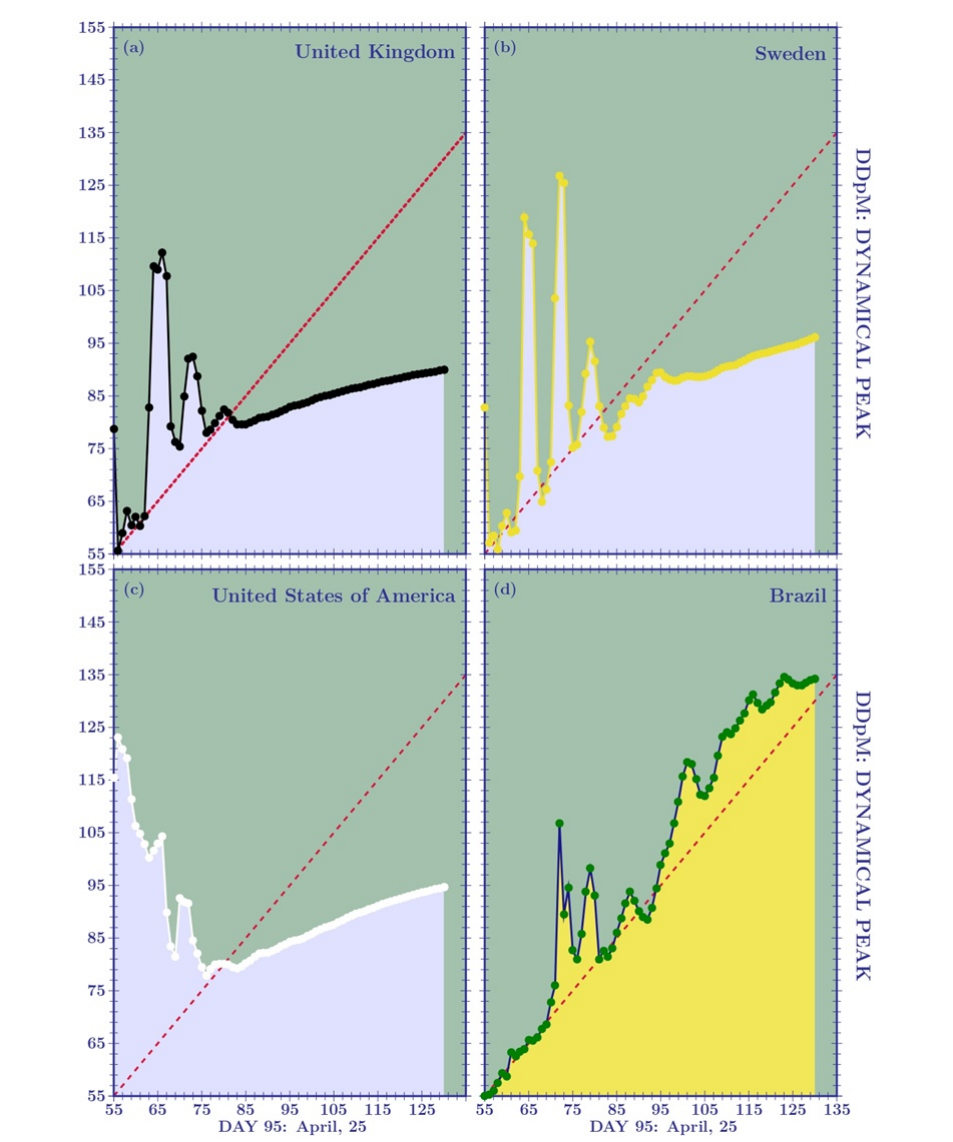
The dynamical curve for the peak of daily deaths per million (DDpM). The oscillatory behavior tends to stabilize when the curve crosses the critical (dashed red) line. After stabilization, the inclination is an indication of the breaking of symmetry in the distribution.

To make some reliable predictions for Brazil, let us examine the dynamical peak (Figure 9). Until the peak is reached, we cannot speak of asymmetric distributions; hence, the standard normal distribution must be used to obtain *dynamical* predictions. The idea behind dynamical predictions is simple: in the initial stage of the disease, the daily updated data lead to forecasts that change drastically from one day to the next. For example, on day 65 (March 26), the peak of the DDpM curves for the United Kingdom, Sweden, and the United States was predicted to occur on day 103 (May 3), day 109 (May 9), and day 116 (May 16), respectively (Figure 9). Five days later, the peak of the DDpM curves was predicted on day 92 (the United Kingdom and the United States) and day 127 (Sweden). In Figure 9, the dashed red line (day of the prediction coinciding with the prediction of the peak) represents the critical line. When the prediction curve crosses such a line, it tends to stabilize (see the United Kingdom, Sweden, and the United States). For Brazil, the oscillating peak is getting closer to the critical line. For a symmetric distribution, after the crossing point, we should, theoretically, have a horizontal line. Therefore, the inclination of the dynamical curve, after the crossing point with the critical line, is an indication of the breaking of symmetry in the distribution. For example, the DDpM skew-normal curves of the United States and Sweden should have greater asymmetry compared to the United Kingdom. This is confirmed by the standard deviations provided in Table 4.

The dynamical analysis of Brazil’s peak shows that the country is approaching its DDpM peak. To see when this will happen, let us consider the number of deaths on May 30 (day 130), that is, 28,834. If we go back to day 80 (April 10), we find 1057 deaths. Table 10 displays the number of deaths every 5 days starting on April 10.

The ratios between the number of deaths every 5 days (eg, 1.64=1736/1057) can be modeled using a linear fit: *yd=*2.4−*xd/*100. Solving for *xd* and setting *yd=*1, we find that *xd*=140, which predicts the peak of the DDpM curve to fall around June 10. Considering the increase of the last 10 days, this indicates a peak of around 200 TDpM, a number comparable to that of the most critical European countries (Figure 3B) but with a number of DDpM at a peak lower than those of these countries (Figure 4B) and similar to the Dutch and Swedish peaks. Recalling that the Netherlands is closing its first pandemic wave at around 400 and the prediction for Sweden is around 500, for Brazil this means approximately 80,000 deaths if the mitigation measures remain similar to the current ones (which are comparable to the Netherlands’ approach). Relaxing the mitigation rules (resembling Sweden’s approach) will probably result in surpassing a TDpM value of 600, that is, exceeding 120,000 deaths.

Looking at the TDpM situation across the five regions of Brazil, we find (on May 31) a very heterogeneous situation, with the Central-West (TDpM=23) and South (TDpM=18) regions well below the national value of 140, the Northeast (TDpM=155) and Southeast (TDpM=157) regions with a TDpM comparable to the national one, and the Northern (TDpM=309) region surpassing the national one. In São Paulo State (TDpM=166), São Paulo City (11.8 million inhabitants) has a TDpM of 357 whereas Campinas (1.2 million inhabitants) has a TDpM of 63. This large heterogeneity indicates the impact of varying local mitigation measures when combating the long epidemic wave.

**Table 10:** The number of deaths every 5 days, starting on April 10, and ratios between the number of deaths every 5 days, for Brazil.

Day	Deaths, n	Ratio
80	1057	N/A^a^
85	1736	1.64
90	2587	1.49
95	4057	1.57
100	6006	1.48
105	7938	1.32
110	11,123	1.40
115	14,962	1.35
120	18,859	1.26
125	23,473	1.24
130	28,834	1.23

^a^N/A: not applicable.

### ρ Factor Analysis

Table 11 presents the *ρ* factor for the 10 European countries of Figures 5 and 6 by using the data in Table 1. A lower value implies a better rating.

Table 12 displays the *ρ* factor for countries that have adopted a smart testing strategy and reduced the number of deaths per 1 million inhabitants at the end of May 2020 [2].

**Table 11:** The ρ factor for 10 European countries.

Country	*ρ* factor
Switzerland	0.80
Ireland	0.86
The Netherlands	0.96
Germany	1.01
Portugal	1.07
Austria	1.08
France	1.16
Spain	1.69
Italy	2.35
Belgium	2.42

**Table 12:** The total deaths per 1 million inhabitants, total confirmed cases per million, tests per confirmed case, population size in millions, and ρ factor on May 31, 2020.

Country	Total deaths per million	Total confirmed cases per million	Tests per confirmed case	Population size in millions	*ρ* factor
Iceland	29	5295	33.8	0.34	0.19
South Africa	12	552	22.2	59.24	0.48
Norway	44	1558	29.1	5.42	0.82
Finland	58	1243	26.9	5.54	1.26
Czech Republic	30	866	47.8	10.71	1.66
South Korea	5	224	80.2	51.27	1.79
Australia	4	283	204.2	25.47	2.89

It is important to recall that the *ρ* factor considers not only the mortality rate but also the *immunization* rate. It is clear that with an indiscriminate and strict lockdown, a country will avoid deaths, but at the same time, it will have a very low immunization level when facing the second wave of the pandemic.

Table 12 is also useful for understanding why the TpC is important. For example, South Africa and South Korea have similar mortality rates: 12/552 and 5/224, respectively. However, South Korea’s testing strategy led to a number of tests 4 times that of South Africa. Consequently, the number of infected people in South Africa is expected to be greater than that of South Korea probably by the same factor. This explains the final ratio of the *ρ* factor between South Africa and South Korea.

In the case of Italy, where a full national lockdown was imposed at the beginning of March, Table 13 presents metrics and the *ρ* factor associated with each of its regions.

**Table 13:** The total deaths per 1 million inhabitants, total confirmed cases per million, tests per confirmed case, population size in millions, and ρ factor for the regions of Italy on May 30, 2020.

Region/country	Total deaths per million	Total confirmed cases per million	Tests per confirmed case	Population size in millions	*ρ* factor
Piedmont	865	6857	10.3	4.46	1.30
Lombardy	1598	8823	8.4	10.06	1.52
Valle d’Aosta	1172	9697	12.7	0.12	1.53
Liguria	941	6226	10.9	1.55	1.65
Molise	71	1406	33.0	0.31	1.67
Emilia-Romagna	921	6224	11.6	4.46	1.72
Marche	645	4397	15.2	1.53	2.23
Trentino-Alto Adige	704	6565	21.6	1.07	2.32
Tuscany	278	2708	24.7	3.73	2.54
Umbria	86	1626	48.9	0.88	2.59
Abruzzo	308	2471	22.6	1.31	2.82
Apulia	124	1114	26.0	4.03	2.89
Lazio	124	1312	32.8	5.88	3.10
Veneto	390	3903	34.4	4.91	3.44
Sicily	55	688	43.3	5.00	3.46
Campania	71	827	41.3	5.80	3.55
Sardinia	79	827	41.7	1.64	3.98
Friuli-Venezia Giulia	273	2681	40.3	1.22	4.10
Basilicata	48	712	73.7	0.56	4.97
Calabria	50	594	59.9	1.95	5.04
Italy	551	3846	16.4	60.47	2.35

From these data, it is clear that regions such as Calabria (TCCpM=594, TpC=59.9), Sicily (TCCpM=688, TpC=43.3), Basilicata (TCCpM=712, TpC=73.7), Sardinia (TCCpM=827, TpC=41.7), and Campania (TCCpM=827, TpC=41.7) have a very low immunization rate; this should be considered when entering the second wave of the pandemic. The best factor, combining the mortality and immunization rates, belongs to Piedmont. The Italian data also show that a smart lockdown and an appropriate testing strategy should provide better results than an indiscriminate full lockdown.

## Discussion

### Principal Findings

In this final section, after studying the metrics associated with the COVID-19 outbreak, we recommend following these steps:

1. The weekly transmission rate of the DCCpM (DDpM) as countries reach the same number of TCCpM (TDpM) can be used to compare countries that are at different stages of the outbreak, which we refer to as the α (or β) factor;
2. Before reaching the peak, the dynamical (oscillatory) curve of the parameters to be fitted can be used to understand when such a curve crosses the critical line and tends to stabilize;
3. After reaching stabilization, asymmetrical distributions have to be introduced to model the DCCpM and DDpM curves (we used skew-normal distributions).

As shown in the previous sections, the timely massive testing strategy implemented by German authorities resulted in a substantial difference in the outcomes of Germany and Italy. Indeed, mitigation measures (such as physical distancing, contact tracing, restricting public gatherings, closing schools and universities) certainly become more effective when a country adopts a timely and massive testing strategy, thereby limiting transmission from asymptomatic cases and facilitating treatment for sick people before the disease worsens. The quantitative impact of a massive testing strategy has been studied by Gorji et al [28]. Clearly, if a country has not performed enough tests, a random smart testing strategy is required. By testing a much smaller number of randomly selected people per day, it is possible to obtain information on the local transmission rate [29].

The Brazilian mitigation measures are similar to that of the Netherlands, stricter than that of Sweden but certainly less severe than the Italian lockdown. On May 30, Brazil reached a TCCpM value of 2347 and a very low TpC number (1.9), suggesting a great number of hidden infected people. Nevertheless, the number of deaths (TDpM=126) still remain under control, and as shown in the *Results* section, the peak may possibly occur around June 10. For Brazil, the factor is 0.10. This means that, at the end of the first pandemic wave, Brazil will reach a great number of confirmed cases per million (with a consequently good level of population immunization) and a relatively low number of deaths. As shown for Italy, it is clear that a strict national mitigation approach is not the correct way to manage the pandemic. A smart local lockdown should be preferred to a national one, as in medieval times. In contrast to most other European countries where people were virtually housebound, the Brazilian, Dutch, and Swedish authorities adopted a different mitigation approach: conservative (but not medieval), moderate, and liberal, respectively. Italy and the Netherlands are closing their first pandemic wave with TDpM and TCCpM numbers of approximately 550 and 3800 for the former and 350 and 2800 for the latter. Sweden, if the predictions are correct, should close around 550 and 7500. The Dutch and Swedish approaches have yielded positive results in terms of deaths and confirmed cases per million compared to the European countries that adopted a strict lockdown (Belgium, Spain, the United Kingdom, and Italy), even though they were heavily criticized in the beginning for their mitigation measures and despite their less effective testing strategies.

Alarming predictions of the exponential growth rate of the pandemic led the local authorities of many countries to implement a strict lockdown. Nevertheless, the Swedish DCCpM curve does not confirm this fear, and it has a smooth increase with respect to the curves of the United Kingdom and the United States (Figure 7). Recently, Norwegian authorities have concluded that the virus was never spreading as quickly as predicted and that the effective reproduction rate had already dropped to a value around 1.1 before the implementation of most rigid mitigation measures [30]. This is also happening for Brazil (Figure 4A), where starting from day 80 (April 10) and reaching day 130 (May 30), we have, every 5 days, an increase of 1.30-1.45 in the total number of confirmed cases.

### Need for a Massive Testing Strategy

Testing far more people means detecting more inhabitants with fewer or no symptoms. Increasing the number of known cases, but not the number of fatalities, we obviously decrease the fatality rate and obtain a more reliable number for the mortality rate of the pandemic. Nevertheless, this is not the main goal of a massive testing strategy. The strategy of early and widespread testing allows us to slow down the pandemic spread by isolating known cases while they are infectious and to deliver medical treatment in a timelier fashion, thereby saving lives. The possibility of an early diagnosis, before the health of a patient declines substantially, increases the chance of survival.

Long before recording its first case of COVID-19 in February, Germany, in mid-January, developed a test and posted the formula online, and laboratories across the country stockpiled test kits [31]. This permitted greater testing with respect to other European countries. The German and Austrian massive testing strategy, implemented during the early stage of the pandemic, made a great difference. Massive testing in the final stage is only useful for reducing the mortality rate on paper and not for saving a *substantial number of lives.*

At the beginning of its outbreak, Germany conducted 120 tests per confirmed case, far more than any other European country. Medical staff, who were at heightened risk of contracting and spreading the virus, were regularly tested. Donning adequate protection, physicians, nurses, and laboratory technicians took to the streets, conducting tests via the *corona taxi* and suggesting hospitalization even for patients with mild symptoms [31]. This was done at zero cost to the population (contrary, for example, to what happened during the first several weeks of the outbreak in the United States), and this guaranteed broad-based testing. In most countries, including the United States and Brazil, testing was largely limited to the sickest patients. Testing and tracking was a successful strategy used by both South Korea and Germany.

Social distancing measures are important for flattening the pandemic curve and avoiding the collapse of national health care systems. Clear, detailed, and scientifically correct information is fundamental to reassure and calm citizens, but, as already mentioned, massive testing strategies make a noticeable difference in the fight against COVID-19.

An important consideration must be made about the absolute numbers often used in the media: they cannot be used when comparing different countries. For example, the absolute numbers of tests, on May 30, for Germany and Italy, are 3,824,621 and 3,952,971, respectively. At first glance, the small difference seems not to deserve a deep analysis of their strategy. However, as shown in this section, the massive testing strategy adopted by Germany in the early stage of the disease led to different results in terms of mortality rates, in favor of the German people.

Other absolute numbers often used to compare countries are total confirmed cases and total deaths. For example, in the COVID-19 world ranking on Worldometer [2] (which lists 215 countries), the absolute numbers for total confirmed cases and total deaths for Brazil on May 30 puts the country in position 2 for TCCpM (after the United States) and in position 4 for TDpM (after the United States, the United Kingdom, and Italy). To compare countries, we obviously have to normalize using their population; upon normalization, Brazil descends to position 39 for TCCpM and 22 for TDpM.

### Conclusions

We conclude by noting that this paper only represents one of the many different ways of examining numerical data pertaining to the COVID-19 outbreak. Any scientific analysis should always be complemented by examining the local situation in terms of ICU beds, hospital capacity, and equipment. Researchers working with these data can certainly shed some light on the situation, but nurses and physicians struggle on a daily basis to help the population; they save lives, deserve protection, and all the necessary support.

Comparing the epidemic across various countries certainly is a difficult task. Mortality rates must always be traced back to the average age of the population, to the capacity of the health system, and to the strategies adopted by the authorities to manage the COVID-19 outbreak. The discussion and statistical analysis presented in this paper clearly show why Germany was so effective in pandemic management compared Italy. Massive testing strategies are a more appropriate way to control the pandemic. Skew-normal distributions allow us to obtain a more realistic prediction of the end of the pandemic in each country. The mortality rate has to be calculated by comparing the deaths in 2020 with those of 2019; this is the only effective way to understand the effect of COVID-19 on the mortality rate of a country and consequently to understand the real mortality rate associated with the disease and whether deaths were due to overloaded health care systems.

## Appendix

Multimedia Appendix 1 Illustration of normal and skew-normal distributions.

## Abbreviations

DCCpM: daily confirmed cases per million
DDpM: daily deaths per million
EF: effectiveness factor
ICU: intensive care unit
R_0_: basic reproduction number
R_t_: effective reproduction number
SARS: severe acute respiratory syndrome
TCCpM: total confirmed cases per million
TDpM: total deaths per million
TpC: tests per confirmed case
WHO: World Health Organization
