# Supplementary figures and images for: Impact of COVID-19 Testing Strategies and Lockdowns on Disease Management Across Europe, South America, and the United States: Analysis Using Skew-Normal Distributions
Source: JMIRx Med. 2021 Apr 21;2(2):e21269. doi: 10.2196/21269 (PMC8086775; doi:10.2196/21269)

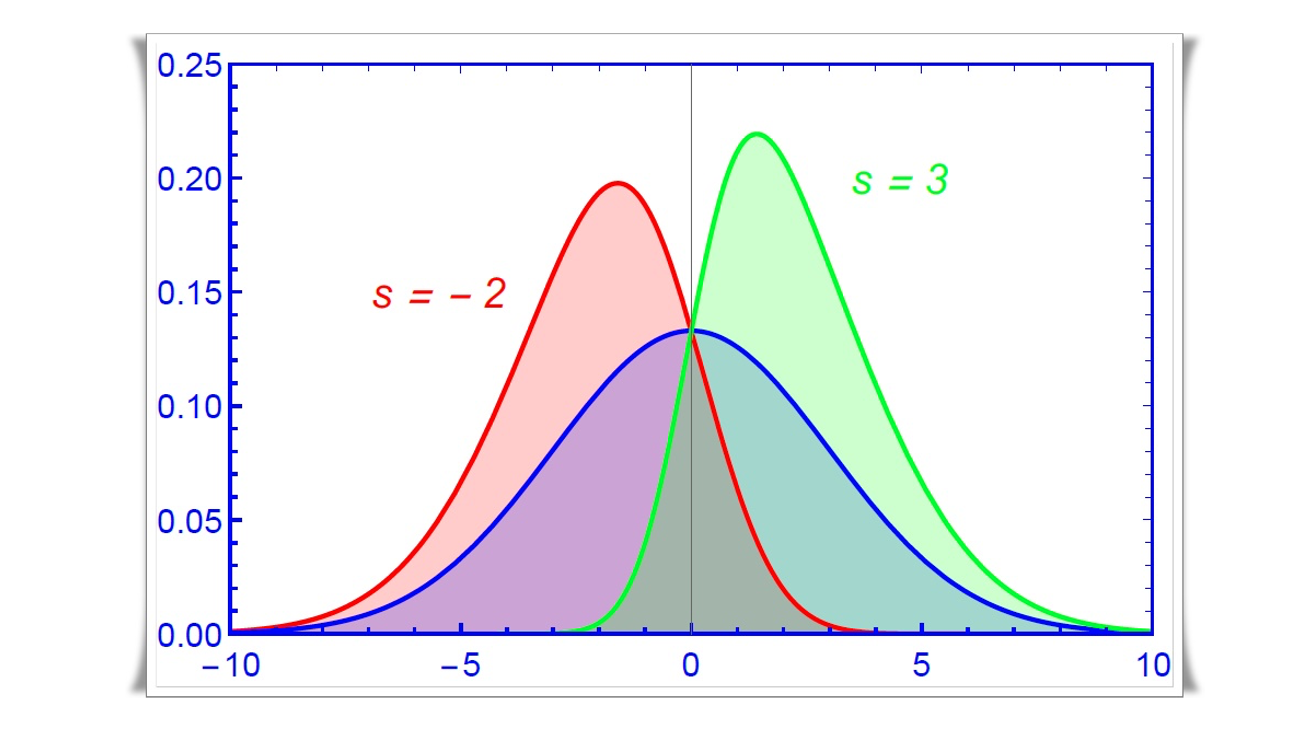

Supplement: Multimedia Appendix 1 [file xmed_v2i2e21269_app1.png]
